# Reliability of a Caton-Deschamps-derived patella height index for knee arthroplasty

**DOI:** 10.1007/s00264-020-04931-0

**Published:** 2021-01-20

**Authors:** Christian Konrads, Lucia C. Grosse, Sufian S. Ahmad, Fabian Springer, Anna J. Schreiner, Florian Schmidutz, Felix Erne

**Affiliations:** 1 Department for Trauma and Reconstructive Surgery, BG Klinik, University of Tübingen, Tübingen, Germany; 2 Center for Musculoskeletal Surgery, Charité - University Medical Center Berlin, Berlin, Germany; 3 Department of Radiology, BG Klinik, University of Tübingen, Tübingen, Germany; 4 Department of Orthopaedic Surgery, Physical Medicine and Rehabilitation, University of Munich (LMU), Munich, Germany

**Keywords:** TKA, Patella height, Patella infera, Patella baja, Anterior knee pain, Insall-Salvati

## Abstract

**Purpose:**

The original Caton-Deschamps index (oCDI) detects functional patella height. It cannot be used in knees with an implanted endoprosthesis. The “modified Caton-Deschamps index” (mCDI) for knee arthroplasty can miss pseudo-patella-infera (PPI), which is common after TKA. A derivate of the oCDI could be a simple analogue to the index published in 1982 using a modified tibial reference point at the anterior proximal point of the inlay, which can indirectly be located on the lateral knee radiograph. It was the aim of this study to determine the intra- and inter-rater agreement of a derived Caton-Deschamps index (dCDI) for knee arthroplasty. We hypothesized that the derived Caton-Deschamps index (dCDI) is a reliable radiological measure for patella height in knee arthroplasty.

**Methods:**

Several patella height indices were measured by three independent raters in two passes. The second pass was performed after 6 weeks in random order. Intra- and inter-observer agreements were determined and analyzed using the intraclass correlation coefficient (ICC). For radiographic evaluation, digital lateral radiographs of 150 knees before and after primary TKA were used.

**Results:**

We found high interrater reliability for all analyzed indices. We found the highest agreements for the ISI preop (ICC = 0.914) and postop (ICC = 0.920), respectively. We also found very good intra-rater reliability for the CDI (ICCpreop = 0.954), dCDI (ICCpostop = 0.945), ISI (ICCpreop = 0.960; ICCpostop=0.940) and BPI (ICCpreop = 0.969; ICCpostop = 0.955). Fourteen cases (9.3%) with insignificant PPI were found.

**Conclusion:**

The derived Caton-Deschamps index (dCDI) can easily be used in knee arthroplasty and demonstrated high intra- and interrater agreement, which was similar to other commonly used and established patella height indices.

## Introduction

Measuring patella height has been a hot topic since decades [1–4]. Multiple radiological indices exist for native knees and for arthroplasty [5–7]. True patella height is determined by the length of the patellar ligament. Pseudo patella height alteration is a relative alteration of the patella height in relation to the femoro-tibial joint line without change in the length of the patellar ligament [8]. Proximalisation of the joint line can be accidentally created during total knee arthroplasty (TKA) leading to pseudo-patella-infera (PPI) [9–11]. Patella infera can be a combination of TPI and PPI. Both components should be part of a complete patella height assessment, which demands a combination of patella height indices [12–14]. If only one index is to be used, it should be made sure that this index can completely detect the sought-after patella height pathology.

The Insall-Salvati index (ISI) detects true patella height only [15]. The original Caton-Deschamps index (CDI) published in 1982 is commonly accepted as a measure of functional patella height [3, 16]. As the tibial landmark used for this index is being resected during knee arthroplasty and as the tibial inlay is not visible on X-ray due to its radiolucency, the index in its original form cannot be used in knees with an implanted endoprosthesis. The “modified Caton-Deschamps index” (mCDI) for knee arthroplasty published in 2016 can miss the detection of PPI. This is a problem as PPI is common after TKA [17].

**Fig. 1:**
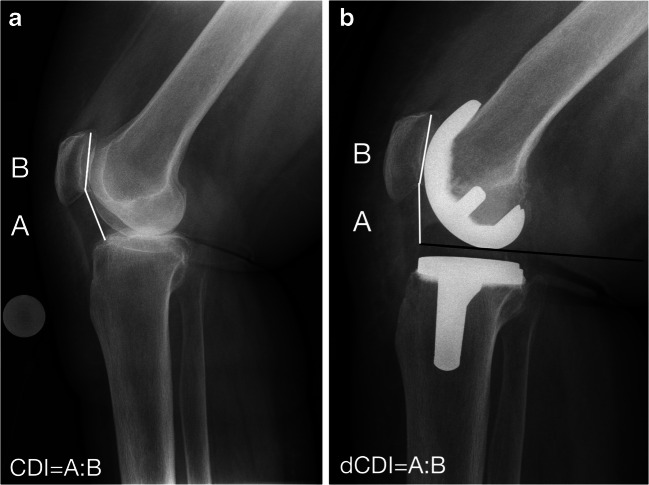
Caton-Deschamps index for native knees (**a**) and derived Caton-Deschamps index for arthroplasty (**b**). CDI = Caton-Deschamps index; dCDI = derived Caton-Deschamps index

A derivate of the original CDI could be a simple analogue to the index published in 1982 using a modified tibial reference point at the anterior proximal point of the inlay, which can indirectly be found on the lateral knee radiograph (Fig. 1). It was the aim of this study to determine the intra- and inter-rater agreement of a derived Caton-Deschamps index (dCDI) for knee arthroplasty.

We hypothesize that the derived Caton-Deschamps index (dCDI) is a reliable radiological measure for patella height in knee arthroplasty.

## Patients and methods

From our database, we retrospectively identified the patients who received a primary bicondylar standard TKA in 2015. Randomly, 150 patients were chosen.

All operations were performed by experienced surgeons and strictly according to internal standard operation procedures. Peri-operatively, an intravenous medication with 2 g Cefazolin was applied as a single shot. All operations were controlled by computer navigation using the Orthopilot TKA software. After median skin incision, an anteromedial parapatellar arthrotomy was performed. The Hoffa fat pad was partially resected. A patellar denervation with resection of osteophytes was done as part of the standard procedure. The rotation of the femoral component was oriented in relation to the transepicondylar axis. Prior to the cemented implantation of tibial and femoral components, the pneumatic tourniquet was applied, and bone surface was prepared by Jet Lavage. In addition to the software protocol, a manual control of range of motion and patella tracking were performed. A closed suction drainage was applied. In preparation of wound closure, local anaesthetics and tranexamic acid were injected. After wound closure, a sterile dressing was applied.

All patients were mobilized with allowed full weight-bearing at the first day after surgery. The suction drainage was removed at the second day after surgery. Patients received physiotherapy daily. Discharge was 7.0 days after surgery. A three week rehab was organized. All patients had a post-operative follow-up appointment about three, six, 12 and 24 months after surgery for clinical and radiographic control.

**Fig. 2:**
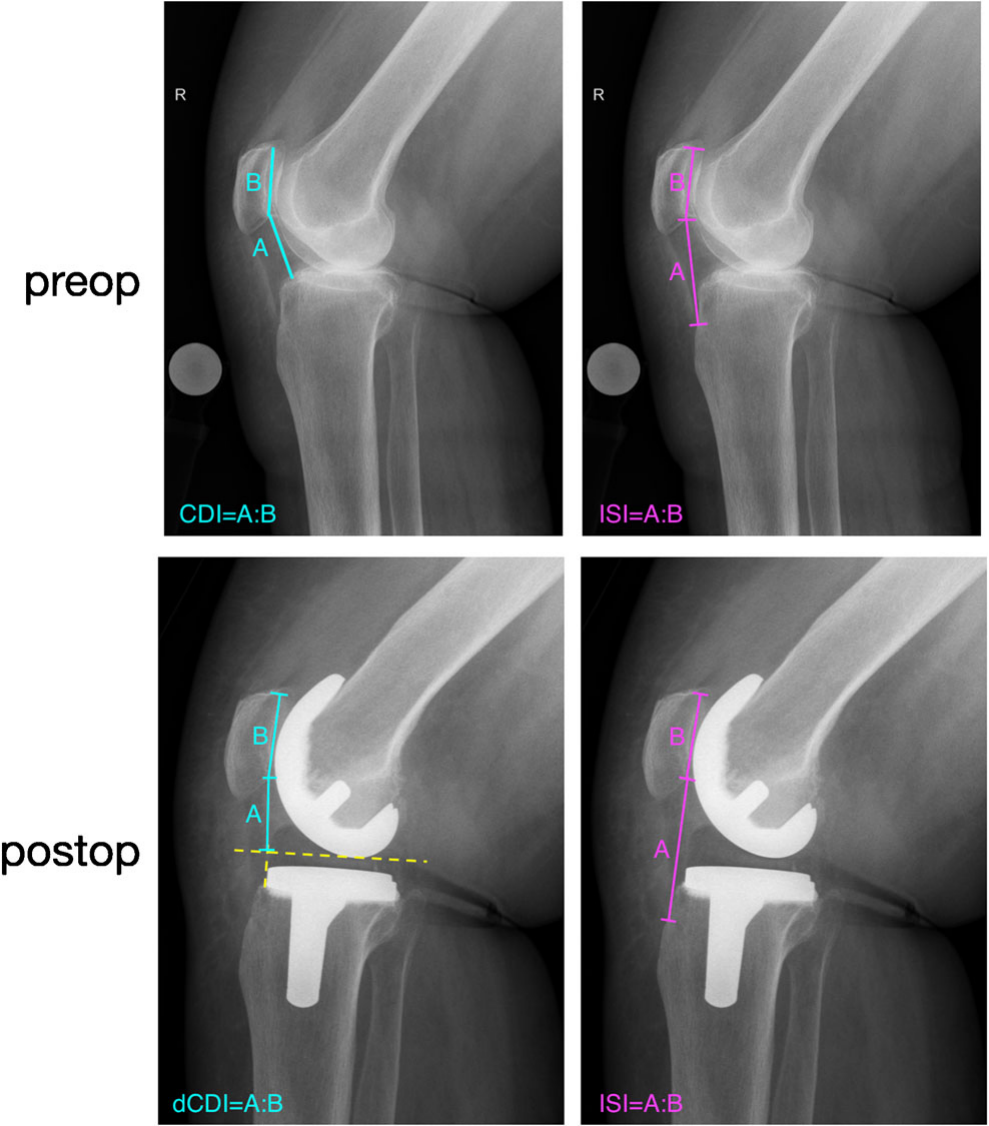
Essential indices for patellar-height analysis determining true patella height and pseudo patella height alterations. ISI = Insall-Salvati index, CDI = Caton-Deschamps index, dCDI = derived Caton-Deschamps index (modification of the CDI for arthroplasty)

We chose the following radiological indices for patella height measurements on the lateral radiographs:

- Insall-Salvati index (ISI) [15, 18]
- Modified Insall-Salvati index (mISI) [19]
- Caton-Deschamps index [16, 20] (CDI) pre-operatively and a derived Caton-Deschamps index [6] (dCDI) post-operatively (Fig. 2)
- Blackburne-Peel index (BPI) [21]

Values for classification into groups of patella infera, norma, or alta are shown in Table 1.

**Table 1:** Patella height indices

Index	Patella infera	Patella norma	Patella alta
Insall-Salvati	< 0.8	0.8–1.2	> 1.2
Modified Insall-Salvati	< 1.2	1.2–2.0	> 2.0
Caton-Deschamps	< 0.6	0.6–1.2	> 1.2
Blackburne-Peel	< 0.6	0.6–1.0	> 1.0

The radiographic indices were measured on lateral radiographs of the knee independently by three raters (one inexperienced but trained rater, one experienced orthopaedic surgeon and one experienced musculoskeletal radiologist). For determination of not only the interrater reliability, but also the intra-rater reliability, all measures were repeated after six weeks. This was done in a randomized order to eliminate any bias from the first reading.

Intra- and inter-observer agreements were determined and analyzed using the intraclass correlation coefficient (ICC).

Ethical approval was received for the conduction of this study by our university’s review board.

For measuring patella height in knee arthroplasty using the *dCDI*, a tibial reference point at the superior anterior part of the inlay was used. Like in the oCDI, this point lies on the femoro-tibial joint line. It is identified on a lateral knee radiograph by drawing a tangential line to the femoral component. This line is parallel to the tibial component (Fig. 1).

For comparison of the dCDI with the mCDI, we additionally measured the mCDI in ten cases with post-operative PPI.

## Results

Out of 150 patients receiving primary TKA, 82 were females and 68 were males. Seventy-eight were right knees and 72 were left knees. The mean age of patients receiving TKA was 73 (52–88).

Testing the reliability of the derived Caton-Deschamps index (dCDI) as a radiological measure for patella height in knee arthroplasty revealed the following results:

We demonstrated high inter-rater reliability for all analyzed indices (Table 2). We found the highest agreements for the ISI preop and postop, respectively. We also found very good intra-rater reliability for the CDI, dCDI, ISI and BPI (Table 3).

**Table 2:** Inter-rater reliability (ICC) for several patella height indices

Rater	CDI before TKA	dCDI after TKA	ISI before TKA	ISI after TKA
Rater 1–rater 2	0.952	0.912	0.952	0.936
Rater 1–rater 3	0.863	0.818	0.893	0.924
Rater 2–rater 3	0.891	0.826	0.898	0.901

*ICC* intraclass correlation coefficient, *CDI* Caton-Deschamps index, *dCDI* derived Caton-Deschamps index, *ISI* Insall-Salvati index, *TKA* total knee arthroplasty

**Table 3:** Intra-rater reliability (ICC) for several patella height indices

Rater	CDI before TKA	dCDI after TKA	ISI before TKA	ISI after TKA	BPI before TKA	BPI after TKA
Rater 1	0.926	0.918	0.956	0.947	0.969	0.955
Rater 2	0.982	0.972	0.965	0.993		

*ICC* intraclass correlation coefficient, *BPI* Blackburne-Peel index, *CDI* Caton-Deschamps index, *dCDI* derived Caton-Deschamps index, *ISI* Insall-Salvati index, *TKA* total knee arthroplasty

Comparing pre-operative and post-operative radiographs, we found TPI, which did not exist pre-operatively, in 2.2% of the cases post-operatively. PPI was found in 9.8% after TKA.

In cases with post-operative PPI, the preop oCDI was 0.76 ± 0.13 and the postop dCDI was 0.54 ± 0.12 on average, whereas the mCDI was 1.14 ± 0.24 pre-operatively and 1.03 ± 0.28 post-operatively.

## Discussion

Patella infera after TKA is a known complication [13, 22]. Direct shortening of the patellar ligament is called TPI and probably occurs due to scarring during the first two years after surgery [22]. It can detoriate clinical outcome leading to anterior knee pain and reduced range of motion. PPI is a common finding after TKA, and within a certain unknown range, it is most likely not correlated with reduced clinical outcome [8, 9].

Given the fact that patella height assessment is under debate since decades and multiple indices for measuring patella height exist, a reliable concept for determining patella height not only in native knees but also in knee arthroplasty is needed [23]. Therefore, we asked the question, if the original Caton-Deschamps index (CDI) could also reliably be used in knee arthroplasty utilizing a modified tibial reference point at the proximal anterior part of the inlay (Fig. 1). This would make comparison of pre-operative and post-operative patella height feasible using the original CDI pre-operatively and a derived CDI after TKA, for recording the full amount of potential patella infera, which could consist of TPI and PPI. The index that we call dCDI has been used before, but to our best knowledge, it has never been completely analyzed regarding its reliability.

We demonstrated high intra- and inter-observer agreement for the dCDI. The reliability of the dCDI was very similar to the most accepted patella height indices: ISI, BPI and CDI (Tables 2 and 3) [23]. So, patella height measured using the CDI preoperatively can now be compared with the post-operative situation after TKA using the dCDI. Normal values for the dCDI should be the same as for the original CDI, but validation remains pending.

An advantage of the dCDI over other indices is that it can fully detect patella height in knee arthroplasty and it can be used very similar to the oCDI, which is utilized in native knees. Therefore, the dCDI fits perfectly in our concept of patella height assessment, which is depicted in Fig. 2. It is easy to use and differentiation between PPI and TPI is possible.

As the dCDI is referenced at the femoro-tibial joint-line, it detects any patella-height alteration, which could happen also in revision TKA with change of any or all implants. In contrast, the mCDI published in 2016 would remain unchanged in cases of revision with inlay exchange to a different height for example.

This study had a number of limitations. First, it was a retrospective design. Second, the number of knees were limited. Third, the number of observers were limited. Despite these limitations, given that the aim of this study was to prove the concept, the authors agreed on the sufficiency of the design chosen in this study.

## Conclusions

The derived Caton-Deschamps index (dCDI) can easily be used in knee arthroplasty and demonstrated high intra- and inter-rater agreement, which was similar to other commonly used and established patella height indices. For functional patella height assessment, the CDI can be used pre-operatively and the dCDI post-operatively. For full analysis of PPI and TPI, the ISI can be used additionally to determine the amount of TPI, while the amount of PPI can then be calculated. The normal range of the dCDI is most likely the same as for the CDI, but this should be verified and validated in a future study with clinical correlation.

## Data Availability

The datasets generated and analyzed during the current study are available from the authors on reasonable request.
